# The control of postoperative pain in elderly patients undergoing total hip replacement under spinal anesthesia

**DOI:** 10.1186/1471-2318-11-S1-A33

**Published:** 2011-08-24

**Authors:** DM Mattiacci, ML Mingione, D Delle Donne, B Lettieri

**Affiliations:** 1 Department of Anaesthesia, Surgical and Emergency Science, Second University of Naples, Italy

## Background

The hip replacement surgery is intended to reduce the intensity of joint pain and improve quality of life in patients with arthritic disease. Advanced age is not a limit to surgical treatment for subarachnoid anesthesia, which is well tolerated by elderly patients.

The aim of our study is to assess how spinal anesthesia with hyperbaric bupivacaine and buprenorphine and the subsequent use of an elastomeric pump can contribute to postoperative pain control in this type of surgery.

## Materials and methods

We studied 42 patients (16M and 26F) ASA Class II and III. All patients underwent anesthesia sub arachnoid (needle 27G Whitacre, L3-L4, lateral decubitus), premedicated intravenously with 0.01 mg / kg of atropine, 2 mg midazolam, 500 ml NaCl 0.9% and monitored throughout the surgery. In group B 14 mg of hyperbaric bupivacaine 0.5% (2.8 ml) was administered while in group B + B 0.5% hyperbaric bupivacaine 12 mg (2.4 ml) was added to 0.06 mg buprenorphine (0.2 ml).

Before the end of the intervention, we administered 30 mg of ketorolac, tramadol 100 mg, 50 mg of ranitidine, ondansetron 4 mg in 100 ml of NaCl. Upon completion an elastomeric pump containing 60 mg of ketorolac, tramadol 200 mg, 100 mg of ranitidine and ondansetron 4 mg (mixture of 60 ml at 2 ml / h for a total of 30 h ) was applied to each patient. All patients were offered a test protocol used on the rating scale VAS.

## Results

**Figure 1:**
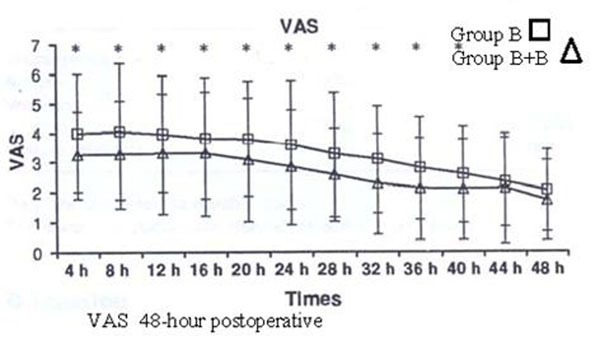


The interventions lasted an average of 157.6 minutes. Recovery times from sensory block was faster in group B (180.2’ vs 205’) while motor block was faster in group B + B (173.4’ vs 169’). In both B and B + B there was a fairly good control of intraoperative hemodynamic and VAS 0. In the 12 postoperative hours the group B + B showed a VAS of 3, less than group B with VAS 6. Table 1 and Figure 1.

**Table 1:** 

Group	Average age	Average weight	Mean Time
B 9M/12W	71.24 (DS+/- 14.43)	70.24 Kg (DS+/- 7.76)	1.66 ‘ (DS+/-0.08)
B+B 7M/14W	74.81 (DS +/-16.66)	73.05 Kg (DS+/- 10.59)	1.66 ‘ (DS+/-0.08)

## Conclusions

The offset of sensory block was longer for group B + B on the administration of buprenorphine. The use of the subarachnoid technique has proved even more effective with the administration of a mixture of hyperbaric bupivacaine and buprenorphine for postoperative pain control with no obvious impact on intraoperative hemodynamic stability, maintaining the same margin of safety of bupivacaine alone.
